# Supplementary material for: Silencing of lncRNA MALAT1 Prevents Inflammatory Injury after Lung Transplant Ischemia-Reperfusion by Downregulation of IL-8 via p300
Source: Mol Ther Nucleic Acids. 2019 May 28;18:285–97. doi: 10.1016/j.omtn.2019.05.009 (PMC6796730; doi:10.1016/j.omtn.2019.05.009)
Supplement: Document S1. Figure S1 [file mmc1.pdf]

OMTN, Volume *18*

Supplemental Information

Silencing of lncRNA MALAT1 Prevents Inflammatory Injury after Lung Transplant Ischemia-Reperfusion by Downregulation of IL-8 via p300

Li Wei, Jiwei Li, Zhijun Han, Zhong Chen, and Quan Zhang

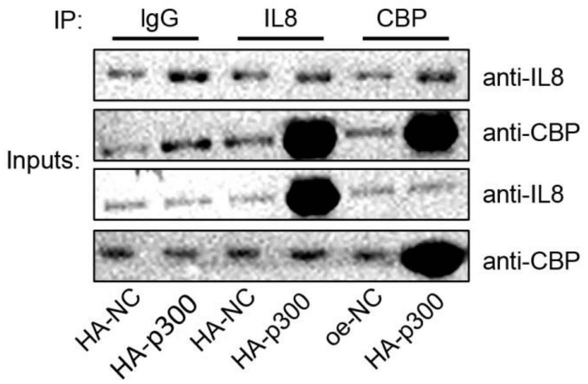

Supplementary Figure 1 The interaction between p300 and IL8 by IP; CBP was used as the positive control while IgG as the negative control.
