# Supplementary material for: Silencing of lncRNA MALAT1 Prevents Inflammatory Injury after Lung Transplant Ischemia-Reperfusion by Downregulation of IL-8 via p300
Source: Mol Ther Nucleic Acids. 2019 May 28;18:285–97. doi: 10.1016/j.omtn.2019.05.009 (PMC6796730; doi:10.1016/j.omtn.2019.05.009)
Supplement: Document S2. Article plus Supplemental Information [file mmc2.pdf]

Molecular Therapy
Nucleic Acids
Original Article

Silencing of lncRNA MALAT1 Prevents Inflammatory Injury after Lung Transplant Ischemia-Reperfusion by Downregulation of IL-8 via p300

Li Wei,[1,2] Jiwei Li,[1,2] Zhijun Han,[1] Zhong Chen,[1] and Quan Zhang[1]

[1]Department of Thoracic Surgery, Henan Provincial People's Hospital, People's Hospital of Zhengzhou University and People's Hospital of Henan University, Zhengzhou 450003, P.R. China

Ischemia-reperfusion injury is a common early complication after lung transplantation. It was reported that long non-coding RNA (lncRNA) metastasis-associated lung adenocarcinoma transcript 1 (MALAT1) is involved in ischemia-reperfusion injury and regulates inflammation. This study aimed to explore the role of MALAT1 in inflammatory injury following lung transplant ischemia-reperfusion (LTIR). A LTIR rat model was successfully established, with the expression of MALAT1 and interleukin-8 (IL-8) in lung tissues detected. Then, *in vitro* loss- and gain-of-function investigations were conducted to evaluate the effect of MALAT1 on pulmonary epithelial cell apoptosis and IL-8 expression. The relationship among MALAT1, p300, and IL-8 was tested. Moreover, a sh-MALAT1-mediated model of LTIR was established *in vivo* to examine inflammatory injury and chemotaxis infiltration. Both IL-8 and MALAT1 were highly expressed in LTIR. MALAT1 interacted with p300 to regulate the IL-8 expression by recruiting p300. Importantly, silencing of MALAT1 inhibited the chemotaxis of neutrophils by downregulating IL-8 expression via binding to p300. Besides, MALAT1 silencing alleviated the inflammatory injury after LTIR by downregulating IL-8 and inhibiting infiltration and activation of neutrophils. Collectively, these results demonstrated that silencing of MALAT1 ameliorated the inflammatory injury after LTIR by inhibiting chemotaxis of neutrophils through p300-mediated downregulation of IL-8, providing clinical insight for LTIR injury.

INTRODUCTION

Lung transplantation is considered one of the effective therapies for patients suffering from end-stage lung disease at final stage, and lung transplantation increased year after year.[1] However, ischemia-reperfusion injury (IRI) remains a severe complication in early lung transplant, and may contribute to the mortality and morbidity induced by lung transplantation and result in development of bronchiolitis obliterans syndrome.[2] Statistical data have shown that 25% patients after lung transplant are affected by IRI, which then leads to major histocompatibility complex class II expression, severe graft dysfunction, and rising mortality.[3] A previous study has reported the risk factors of IRI, such as the preservation techniques, ischemic time of grafts, and the unsuspected donor lung pathology.[4] The therapy for IRI after lung transplantation has always been studied; for instance, Kawamura et al.[5] have revealed that hydrogen inhalation could protect against lung IRI and greatly enhance the function of lung grafts after prolonged cold preservation, transplant, and reperfusion. Although numerous strategies have been conducted to reduce IRI after lung transplantation, the physiopathology of IRI after lung transplantation has not yet been fully elucidated, suggesting that more studies are required for ameliorating the outcomes of IRI after lung transplantation.[6]

Long non-coding RNA (lncRNA) metastasis-associated lung adenocarcinoma transcript 1 (MALAT1), also named as nuclear-enriched abundant transcript 2 (NEAT2), is a highly conserved nuclear non-coding RNA (ncRNA) and is identified as a prognostic biomarker for metastasis and the progression of lung cancer.[7] Previous study has reported that MALAT1 is identified as an essential prognostic factor for recurrence of hepatocellular carcinoma after liver transplantation.[8] Besides, the association between MALAT1 and IRI has been reported in accumulating studies; for instance, MALAT1 is revealed to highly express in patients with acute myocardial infarction, which was correlated with the pathomechanism of myocardial IRI, MALT1 plays crucial roles in renal IRI with regulatory effect on inflammation-induced hyperglycemia in endothelial cells, and MALAT1 displays a cardio-protective role in fentanyl in myocardial IRI.[9–11] Also, MALAT1 has been reported to participate in regulating cardiac inflammation and dysfunction through mediating miR-125b and p38 mitogen-activated protein kinase/nuclear factor κB (MAPK/NF-κB) in sepsis.[12] Interleukin-8 (IL-8), a proinflammatory factor, has been demonstrated to not only regulate activation and migration of neutrophils into tissue from peripheral blood but also serve as chemoattractant and activator of neutrophils in severe inflammation and lung injury.[13] IL-8 also expresses in premalignant epithelial cells in lung cancer, and its high expression relates to an unfavorable

Received 12 April 2019; accepted 17 May 2019;
https://doi.org/10.1016/j.omtn.2019.05.009.

[2]These authors contributed equally to this work.

Correspondence: Jiwei Li, Department of Thoracic Surgery, Henan Provincial People's Hospital, People's Hospital of Zhengzhou University and People's Hospital of Henan University, No. 7, Weiwu Road, Zhengzhou 450003, Henan Province, P.R. China.
E-mail: facefuture00114321@163.com

This is an open access article under the CC BY license (http://creativecommons.org/licenses/by/4.0/).

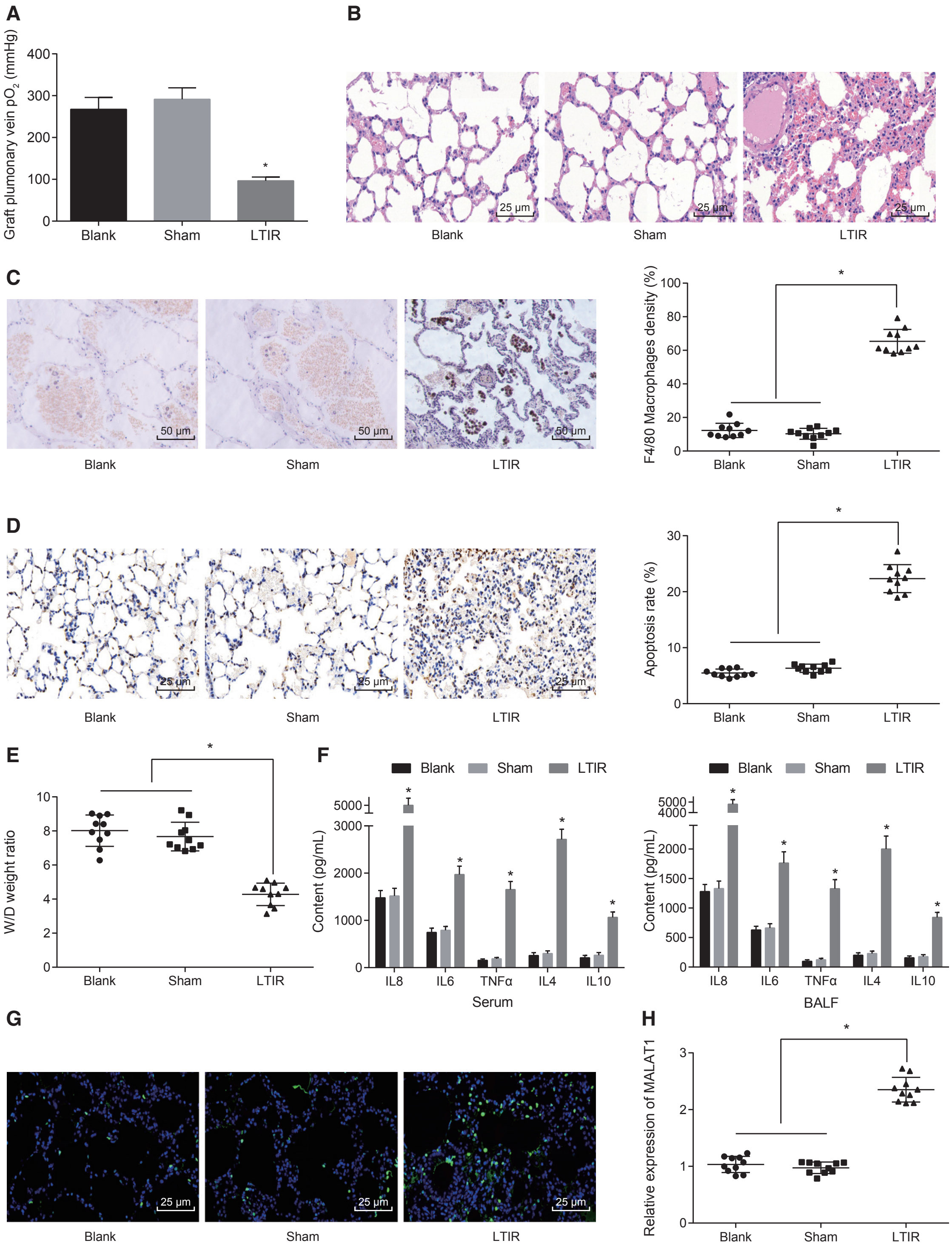

Figure 1. Upregulation of IL-8 and MALAT1 in LTIR Rat Models

(A) The graft pulmonary vena PO_2 after LTIR. (B) The pathological changes of lung tissues after LTIR examined by H&E staining (scale bars, 25 μm; original magnification ×400). (C) The infiltration of macrophages (F4/80) in lung tissues after LTIR examined by IHC (scale bars, 50 μm; original magnification ×200). (D) The apoptosis of pulmonary epithelial cells after LTIR examined by TUNEL assay (scale bars, 25 μm; original magnification ×400). (E) The W/D ratio of lung tissues after LTIR. (F) The expression of IL-6, IL-8, TNF-α, IL-4, and IL-10 in peripheral venous blood and BALF after LTIR. (G) The expression of MALAT1 in lung tissues after LTIR examined by FISH assay (scale bars, 25 μm; original magnification ×400). (H) The expression of MALAT1 in lung tissues after LTIR examined by qRT-PCR. *$p < 0.05$ versus the blank or sham

(legend continued on next page)

prognosis of lung cancer.[14] Additionally, IL-8 was highly expressed in patients after lung transplantation,[15] and inhibition of IL-8 displays a protective role in IRI rat models after kidney transplantation.[16] The histone acetyltransferase p300, a transcriptional coactivator, is essential in various cellular activities including proliferation, apoptosis, and DNA damage response, and its intrinsic activity can regulate chromatin during transcription and other DNA template phenomenon.[17] Importantly, the transcriptional regulation of IL-8 has been reported to participate in chromatin remodeling by histone acetylation.[18] Besides, the association among lncRNA, DNA injury, and histone H4 acetylation has been revealed.[19] Furthermore, neutrophils and neutrophil elastase display significant function in endothelial injury and enhanced vascular permeability, which are the features of acute lung injury,[20] and the effect of cystic fibrosis transmembrane conductance regulator expressed by neutrophils on regulating acute lung inflammation and injury has been reported.[21] Interestingly, Xue et al.[22] demonstrated that lncRNA CMPK2 could promote neutrophils interferon production in systemic lupus erythematodes (SLEs). Based on these findings, we hypothesized that MALAT1 regulated inflammatory injury of rats after lung transplant ischemia-reperfusion (LTIR), and investigated whether IL-8 and p300 are involved in the regulating process.

RESULTS

IL-8 and MALAT1 Are Highly Expressed in the LTIR Rat Model

First, we measured the lung function after transplantation to verify the successful establishment of the LTIR rat model. The results showed that the graft pulmonary vena partial pressure of oxygen (PO_2) was lower in the LTIR group than in the blank and sham groups (Figure 1A). H&E staining revealed that more serious pulmonary alveolar hemorrhage, edema, and interstitial inflammation were observed in the LTIR group than in the blank and sham groups (Figure 1B). The positive expression of macrophages was higher in the LTIR group than in the blank and sham groups (Figure 1C). The number of apoptotic cells was higher in the LTIR group than in the blank and sham groups (Figure 1D). Furthermore, the wet/dry (W/D) ratio increased in the LTIR group compared with the blank and sham groups (Figure 1E). All of the results revealed that the model of LTIR rats was successfully established. Next, the level of inflammatory factors, IL-6, IL-8, tumor necrosis factor alpha (TNF-α), IL-4, and IL-10, in serum was assessed by ELISA, suggesting that IL-8 showed the highest expression in peripheral venous blood and bronchoalveolar lavage fluid (BALF) among all of the tested inflammatory factors (Figure 1F). Also, a close correlation between MALAT1 and an inflammatory reaction has been reported in previous studies;[23,24] we applied fluorescence *in situ* hybridization (FISH) and qRT-PCR to examine the expression of MALAT1, and the results showed that the expression of MALAT1 was significantly higher in the LTIR group than in the blank and sham groups (Figures 1G and 1H).

All of these findings suggested that LTIR causes apparent lung tissue injury, with significantly increased expression of IL-8 in peripheral venous blood and BALF, and increased expression of MALAT1 in lung tissues.

Silencing of MALAT1 Reduces IL-8 Expression and Inhibits Apoptosis of Pulmonary Epithelial Cells

To study the relationship between MALAT1 and IL-8, the expression of IL-8 has been examined in the BEAS-2B cells treated with sh-MALAT1 and overexpressing (oe)-MALAT1. The efficacy of transfection was verified by RT-PCR (Figure 2A). Then, qRT-PCR and ELISA were conducted to detect the mRNA and protein expression of IL-8 in each group. The results revealed that inhibition of MALAT1 significantly decreased mRNA and protein expression of IL-8; in contrast, overexpression of MALAT1 increased mRNA and protein expression of IL-8 (Figures 2B and 2C). Next, cell apoptosis was assessed by flow cytometry, and decreased apoptotic ratio was observed in the sh-MALAT1 group, whereas increased apoptotic ratio was observed in the oe-MALAT1 group (Figure 2D). Subsequently, western blot analysis was conducted to detect the protein expression of apoptosis-related factors, and results revealed that the protein expression of phosphorylated c-Jun N-terminal kinase (p-JNK), phosphorylated P53 (p-P53), and poly-ADP-ribose polymerase (PARP) was apparently decreased in the shMALAT1 group, whereas it was significantly increased in the oe-MALAT1 group (Figure 2E).

Therefore, it could be concluded that silencing of MALAT1 decreased the apoptosis of pulmonary epithelial cells by downregulating IL-8.

MALAT1 Regulates the Expression of IL-8 through Recruiting p300

In order to investigate the relationship between MALAT1 and IL-8, we performed FISH assay, and the results revealed a co-localization of MALAT1 and p300 (Figure 3A), suggesting a potential relationship between MALAT1 and p300. Furthermore, RNA immunoprecipitation (RIP) results indicated that the expression of MALAT1 and the protein expression of p300 were apparently higher in blank and shRNA negative control (sh-NC) groups than in the sh-MALAT1 group (Figure 3B). Then, RNA pull-down and western blot analysis (Figure 3C) suggested that MALAT1 directly bound to p300. Subsequently, qRT-PCR and ELISA were conducted, and the results revealed that the mRNA and protein expression of IL-8 were inhibited by downregulation of p300, whereas they were enhanced by p300 overexpression; besides, the expression of IL-8 was higher in the sh-MALAT1 + oe-p300 group than in the sh-MALAT1 + oe-NC group (Figures 3D and 3E). Additionally, according to the immunoprecipitation (IP) assay results, p300 did not bind to IL-8, suggesting that the expression of IL-8 was regulated by MALAT1 instead of p300 (Figure S1).

group. All experiments were repeated three times. n = 10 in each group. Measurement data were expressed as mean ± SD. Comparisons among multiple groups were analyzed using one-way ANOVA, followed by Tukey's post hoc test. BALF, bronchoalveolar lavage fluid; FISH, fluorescence *in situ* hybridization; IHC, immunohistochemistry; IL-8, interleukin-8; LTIR, lung transplant ischemia-reperfusion; MALAT1, metastasis-associated lung adenocarcinoma transcript 1; PO_2, partial pressure of oxygen; TNF-α, tumor necrosis factor alpha; TUNEL, terminal deoxynucleotidyl transferase (TdT) dUTP nick-end labeling; W/D, weight/dry.

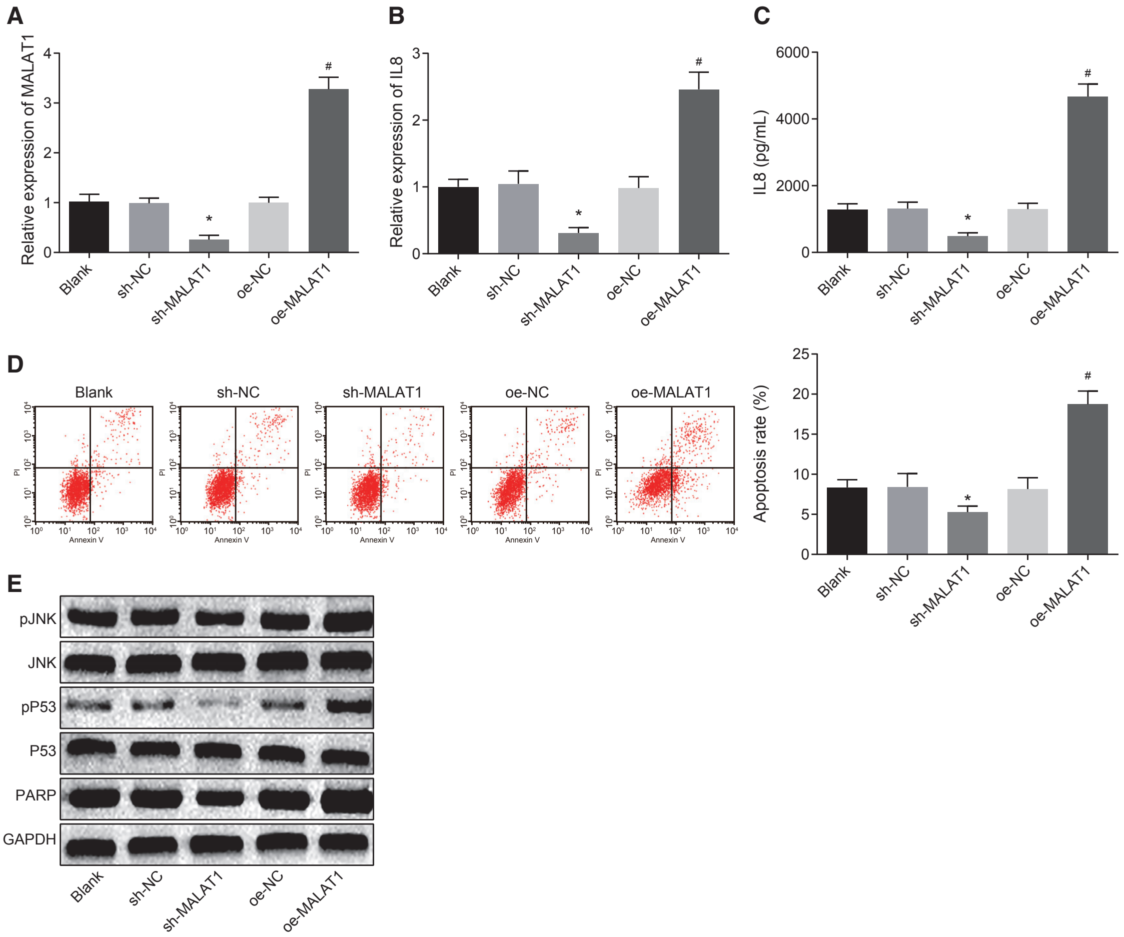

Figure 2. Silencing of MALAT1 Suppresses Apoptosis of Pulmonary Epithelial Cells through Inhibiting the Expression of IL-8

(A) The expression of MALAT1 in pulmonary epithelial cells after transfection. (B) The mRNA expression of IL-8 in pulmonary epithelial cells after transfection, examined by qRT-PCR. (C) The protein expression of IL-8 in pulmonary epithelial cells examined by ELISA. (D) The apoptosis of pulmonary epithelial cells in each group examined by flow cytometry. (E) The protein expression of apoptosis-related factors (JNK, p-JNK, P53, Pp53, PARP) in pulmonary epithelial cells and GAPDH acts as an internal control. *$p < 0.05$ versus the blank or sh-NC groups; $^{\#}p < 0.05$ versus the blank or oe-NC groups. All experiments were repeated three times. n = 10 in each group. Measurement data were expressed as mean ± SD. Comparisons among multiple groups were analyzed using one-way ANOVA, followed by Tukey's post hoc test. GAPDH, glyceraldehyde-3-phosphate dehydrogenase; IL-8, interleukin-8; JNK, c-Jun N-terminal kinase; MALAT1, metastasis-associated lung adenocarcinoma transcript 1; PARP, poly-ADP-ribose polymerase; p-JNK, phosphorylated c-Jun N-terminal kinase.

All of these results indicated that MALAT1 bound to p300 in pulmonary epithelial cells and regulated the expression of IL-8.

p300 Promotes the Activation of IL-8 Transcription through Mediating H3K27ac

To further explore the downstream mechanism that MALAT1 regulated the expression of IL-8, we applied bioinformatics analysis and found H3K27 acetylation (H3K27ac) highly enriched in the IL-8 gene promoter region (Figure 4A). Then, chromatin immunoprecipitation (ChIP) was conducted using P300 or H3K27ac antibody, and the results exhibited higher enrichment of p300 and H3K27ac in the oe-p300 group than in the blank and oe-NC groups (Figures 4B and 4C), which suggested that overexpression of p300 elevated the enrichment of H3K27ac and p300 on the IL-8 promoter region. Furthermore, in order to investigate that the expression of IL-8 was affected by the p300-induced enrichment of H3K27ac in the IL-8 promoter region, the expression of IL-8 was detected in the cells of the oe-NC and oe-p300 with or without p300 inhibitor C646 treatment, and ChIP was conducted using H3K27ac antibody to detect the degree of acetylation. The results exhibited that the DNA enrichment of the IL-8 promoter was significantly reduced in the oe-p300-c646 group compared with the oe-p300 and oe-NC groups (Figure 4D). Besides, ELISA and western blot analysis demonstrated that the expression of IL-8 was significantly decreased in cells of the oe-p300-c646 group compared with the oe-p300 and oe-NC groups (Figure 4E).

These findings suggested that p300 enriched in the IL-8 promoter region and activated IL-8 transcription through regulating H3K27ac.

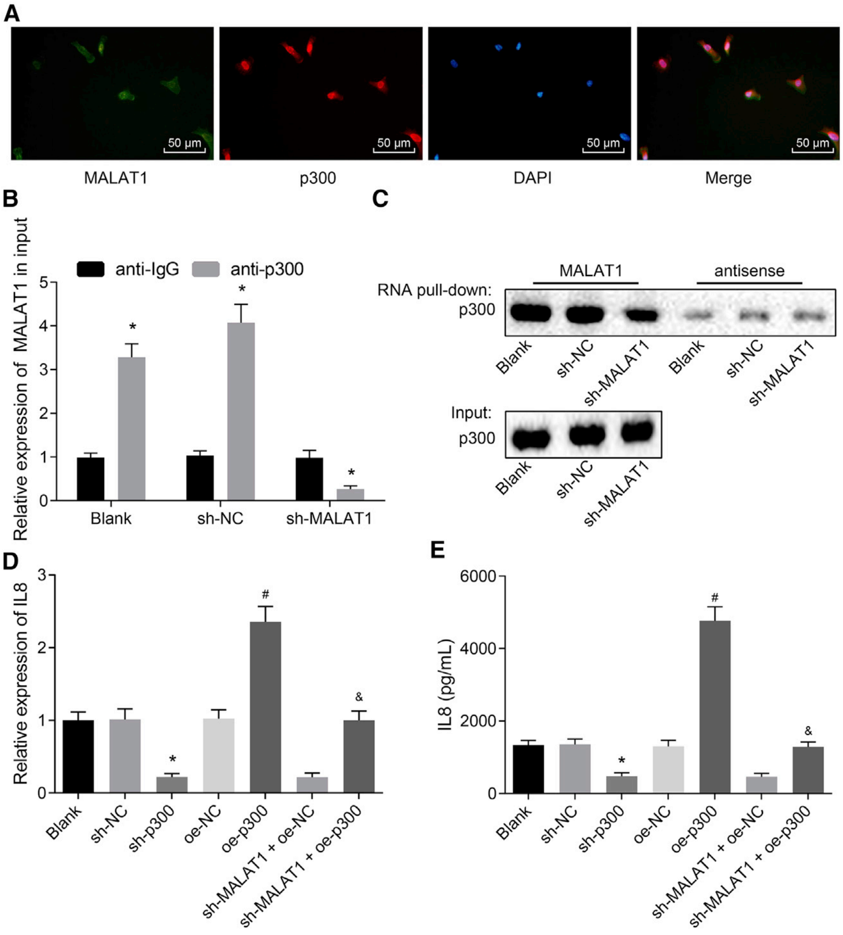

Figure 3. MALAT1 Regulates the IL-8 Expression through Binding to p300 in Pulmonary Epithelial Cells of the LTIR Rat Model

(A) The co-location of MALAT1 and p300 in pulmonary epithelial cells examined by RNA-FISH (scale bars, 50 μm; original magnification ×200). (B) The relationship between p300 and MALAT1 examined by RIP; *p < 0.05 versus the IgG group. (C) The relationship between p300 and MALAT1 verified by RNA pull-down. (D) The mRNA expression of IL-8 in pulmonary epithelial cells after various transfections examined by qRT-PCR. (E) The protein expression of IL-8 in pulmonary epithelial cells after various transfections detected by ELISA. *p < 0.05 versus the blank or sh-NC groups; #p < 0.05 versus the blank or oe-NC groups; &p < 0.05 versus the sh-MALAT1 + oe-NC group. All experiments were repeated three times. Measurement data were expressed as mean ± SD. Comparisons among multiple groups were analyzed using one-way ANOVA, followed by Tukey's post hoc test. FISH, fluorescence *in situ* hybridization; IL-8, interleukin-8; LTIR, lung transplant ischemia-reperfusion; MALAT1, metastasis-associated lung adenocarcinoma transcript 1; RIP, RNA immunoprecipitation.

Silencing of MALAT1 Suppresses the Chemotaxis and Activation of Neutrophils through Regulating p300-Mediated IL-8 Expression

It has been reported that IL-8 is closely related to chemotaxis of neutrophils; in this study, we assessed the expression of IL-8 in pulmonary epithelial cells treated with sh-NC, sh-MALAT1, oe-NC, and oe-P300, and then further examined the chemotaxis of neutrophils after treating with cells culture from various transfections. Western blot analysis results showed that recombinant human IL-8 (rhIL-8) increased the expression of IL-8, p-S6K1, p-Akt, and phosphorylated extracellular signal-related kinases 1 and 2 (p-ERK1/2) in neutrophils; inhibition of MALAT1 reduced the expression of IL-8, p-S6K1, p-Akt, and p-ERK1/2; overexpression of p300 showed the opposite effect; and overexpression of p300 reversed the inhibitory effect caused by downregulation of MALAT1 (Figure 5A). Moreover, the activity of myeloperoxidase (MPO) was detected in neutrophils treated with cell culture after transfection. As shown in Figure 5B, the activity of MPO was reduced by downregulation of MALAT1 but increased by upregulation of p300, and overexpression of p300 can restore the activity of MPO decreased by downregulation of MALAT1 (Figure 5B). Furthermore, Transwell assay results exhibited that migration of neutrophils was inhibited in the sh-MALAT1 group, whereas overexpression of p300 promoted migration of neutrophils and restored migration of neutrophils in the sh-MALAT1-oe-p300 group (Figure 5C). The extracellular reactive oxygen species (ROS) of neutrophils in each group was detected; the effect was the same as activity of MPO (Figure 5D).

Taking all of these results together, it can be suggested that downregulation of MALAT1 inhibited the chemotaxis and activation of neutrophils through downregulating the expression of p300 and IL-8.

Silencing of MALAT1 Ameliorates the Inflammatory Injury after LTIR by Downregulating IL-8 and Inhibiting Infiltration and Activation of Neutrophils

To further test the effect of MALAT1 on inflammatory injury after LTIR *in vivo*, the LTIR rat model infected by adenovirus-mediated sh-MALAT1 was injected with sh-NC and sh-MALAT1 adenoviruses via tail vein. Initially, qRT-PCR was conducted to verify the MALAT1 expression. The results suggested that MALAT1was remarkably downregulated in the sh-MALAT1 group compared with the sh-NC group (Figure 6A), suggesting the successful establishment of a sh-MALAT1-mediated model of LTIR rats. The pulmonary venous blood gas analysis showed that the graft pulmonary vena PO_2 was lower in the sh-MALAT1 group than in the blank and sham groups (Figure 6B). Then, H&E staining showed the lung injury was reduced in the sh-MALAT1 group compared with the sh-NC group (Figure 6C). Besides, the number of positive cells in macrophages was significantly lower in the sh-MALAT1 group than in the sh-NC group (Figure 6D). Relative to the sh-NC group, the W/D ratio was dramatically increased in the sh-MALAT1 group (Figure 6E). Moreover,

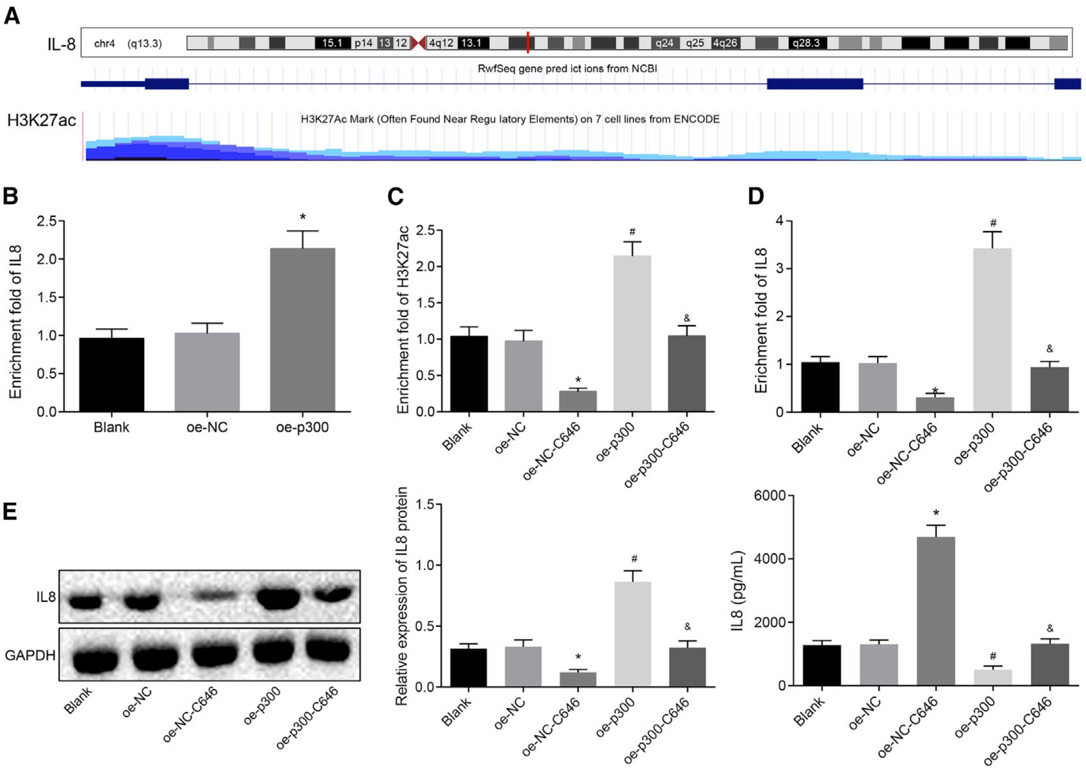

Figure 4. p300 Promotes the Activation of IL-8 Transcription through Mediating H3K27ac

(A) H3K27 acetylation (H3K27ac) highly enriched in the IL-8 promoter region predicted by bioinformatics analysis (http://genome.ucsc.edu). (B) The enrichment of p300 in the IL-8 promoter examined by ChIP. (C) The enrichment of H3K27ac in the IL-8 promoter examined by ChIP. (D) The mRNA expression of IL-8 detected by qRT-PCR. (E) The protein expression of IL-8 examined by ELISA and western blot analysis. *p < 0.05 versus the blank or oe-NC groups; #p < 0.05 versus the oe-NC-c646 groups; &p < 0.05 versus the oe-p300 group. All experiments were repeated three times. Measurement data were expressed as mean ± SD. Comparisons among multiple groups were analyzed using one-way ANOVA, followed by Tukey's post hoc test. ChIP, chromatin immunoprecipitation; H3K27ac, H3K27 acetylation; IL-8, interleukin-8.

ELISA revealed that the expression of IL-8 was significantly lower in the sh-MALAT1 group than in the sh-NC group (Figure 6F). Furthermore, immunohistochemistry (IHC) results exhibited that expression of MPO in the sh-MALAT1 group decreased compared with the sh-NC group (Figure 6G).

All of these results indicated that silencing of MALAT1 could decrease the expression of IL-8 and reduce the infiltration and activation of neutrophils, thus alleviating the inflammatory injury in LTIR rats.

DISCUSSION

IRI following lung transplantation is a serious clinical problem due to its tendency to result in primary graft dysfunction (PGD), and no clinical therapeutic agents have been widely applied in preventing IRI, nor effective treatment strategies.[25] IRI refers to a complicated pathological activity;[6] therefore, understanding mechanisms of IRI is critical for development of novel and effective therapeutic approaches. It has been proved that MALAT1 served as an essential prognostic biomarker for hepatocellular carcinoma after liver transplantation.[8] Besides, IL-8 serves as a major chemo-attractive that plays a vital role in neutrophils chemotaxis, and IL-8 participated in IRI of the rat model following kidney transplantation.[16] Therefore, we hypothesized that MALAT1 could regulate the IRI following lung transplantation through chemotaxis of neutrophils via mediating IL-8. Our results demonstrated that silencing of MALAT1 could reduce the inflammatory injury following LTIR by inhibiting neutrophils chemotaxis through p330-mediated downregulation of IL-8, which provides clinical therapeutic insight for LTIR injury.

It has been revealed that MALAT1 highly expresses in myocardial IRI,[10] and was also found in non-small cell lung cancer with its upregulation related to high metastasis and unfavorable patient prognosis in various diseases.[26] IL-8 also exhibited increased expression in patients occurred inflammation reaction and PGD after lung transplantation.[15] Our results also indicated that MALAT1 and IL-8 were highly expressed in the LTIR rat model. Moreover, we found that MALAT1 could regulate the expression of IL-8, and downregulation of MALAT1 inhibited the apoptosis of pulmonary epithelial

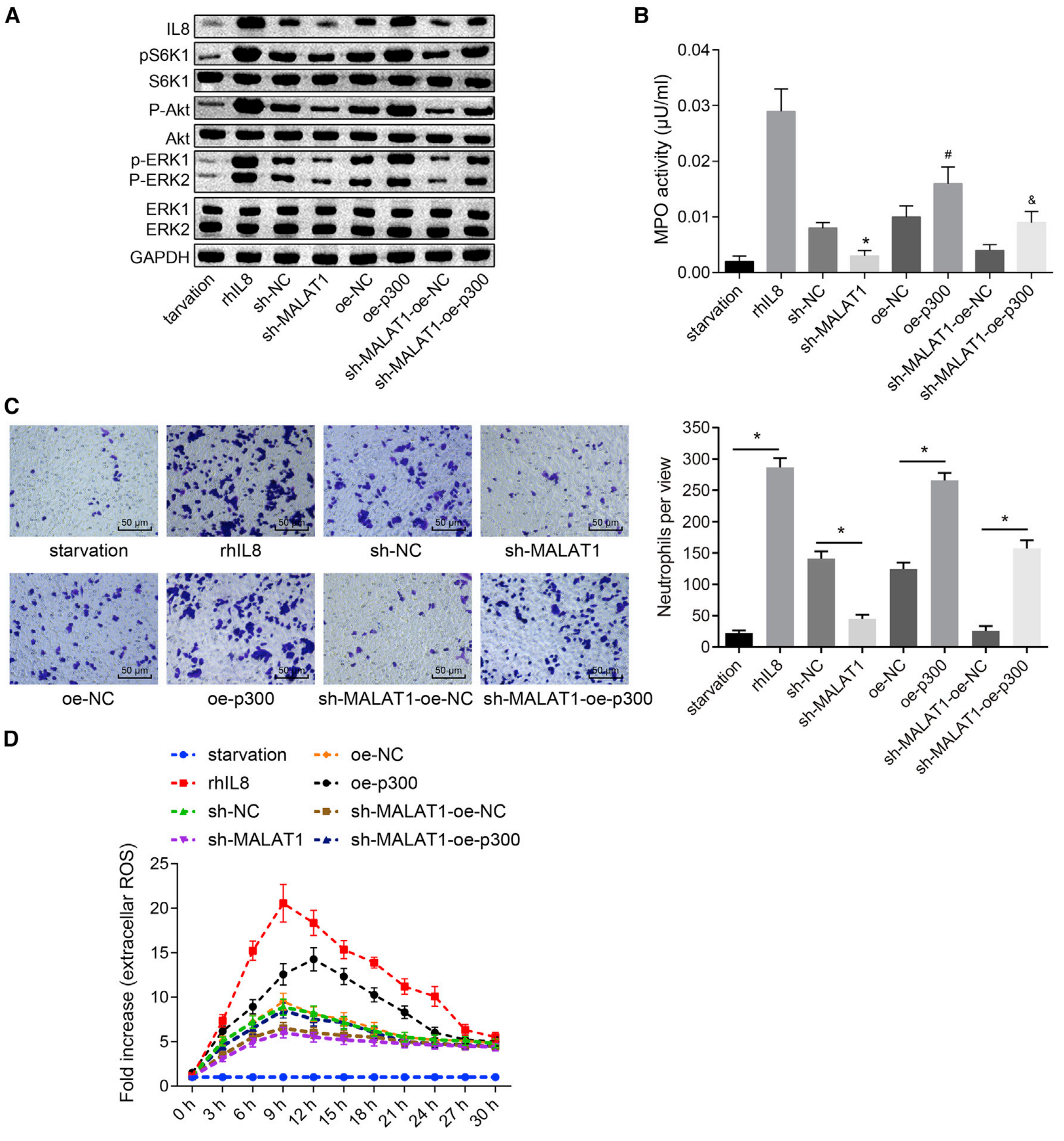

Figure 5. Silencing of MALAT1 Suppresses the Chemotaxis and Activation of Neutrophils through Regulating p300-Mediated IL-8 Expression

(A) The expression of IL-8 and the expression of S6K1, p-Akt, and ERK1/2 in neutrophils in each group (starvation, blank group; recombinant human IL-8 [rhIL-8] protein) examined by western blot analysis. (B) The activity of MPO in neutrophils detected by chromatometry. (C) The migration of neutrophils in each group detected by Transwell assay (scale bar, 50 μm; original magnification ×200). (D) The extracellular ROS of neutrophils in each group. $^{*}p < 0.05$ versus the sh-NC group; $^{\#}p < 0.05$ versus the oe-NC group; $^{\&}p < 0.05$ versus the sh-MALAT1-oe-NC group. All experiments were repeated three times. Measurement data were expressed as mean ± SD. Comparisons among multiple groups were analyzed using one-way ANOVA, followed by Tukey's post hoc test. The data obtained by multiple measurements at different time points were analyzed using repeated-measures ANVOA followed by Bonferroni post hoc test. ERK, extracellular signal-related kinase; IL-8, interleukin-8; MALAT1, metastasis-associated lung adenocarcinoma transcript 1; MPO, myeloperoxidase; ROS, reactive oxygen species.

cells, evidenced by decreased levels of p-JNK, p-P53, and PARP. Similarly, a previous study has reported that lncRNA NEAT1 enhanced the expression of antiviral genes including IL-8.[27] Silencing of MALAT1 suppresses the activation of several factors including JNK1, overexpressed MALAT1 results in their phosphorylation,[28] and MALAT1 could regulate or influence the activity and expression of p53 in disease related to lung.[29,30] Additionally, MALAT1 overexpression suppressed cell apoptosis in bronchopulmonary dysplasia.[31]

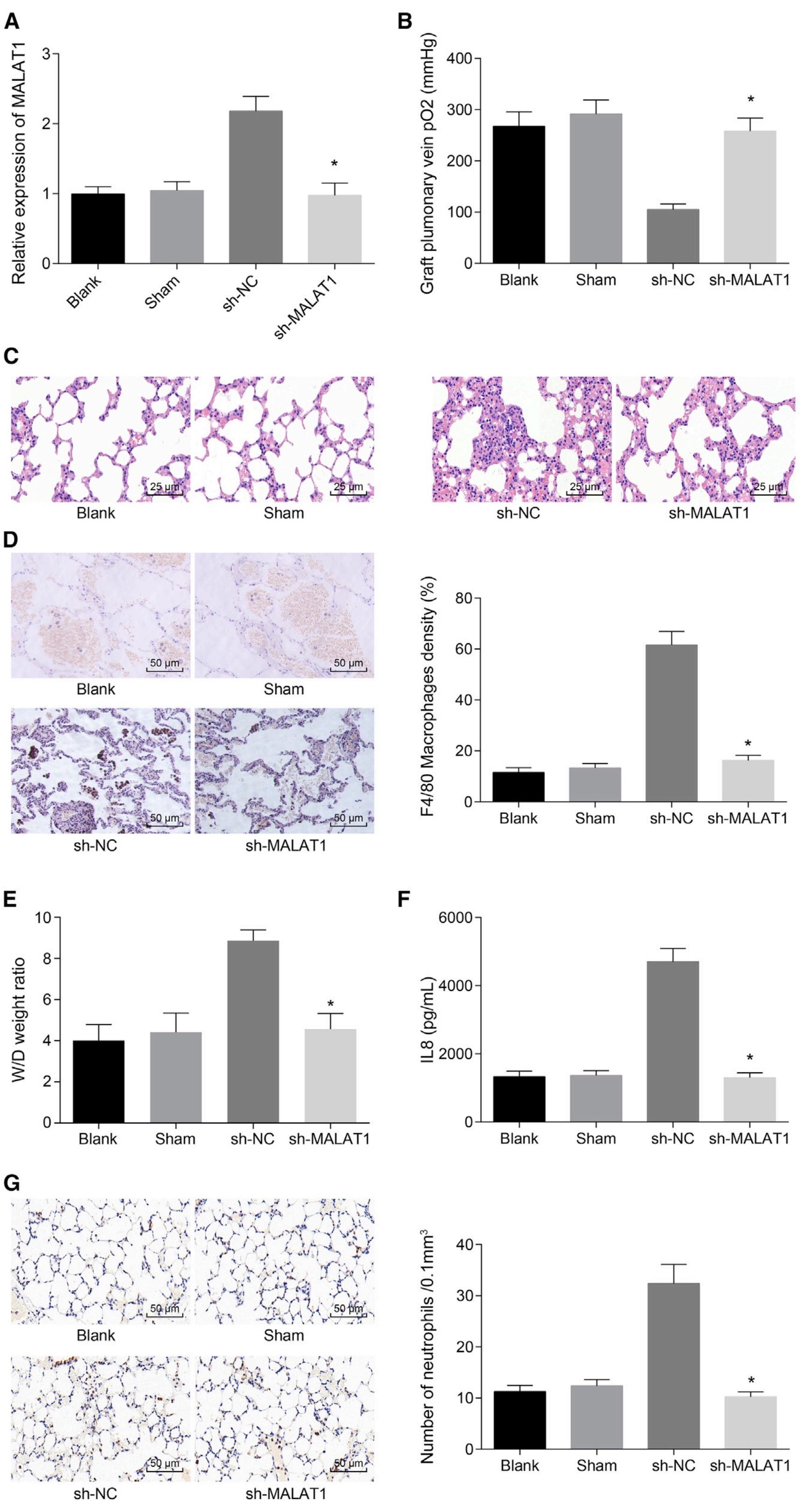

Figure 6. Silencing of MALAT1 Alleviates the Inflammatory Injury after LTIR through Reducing IL-8 Expression and Suppressing Infiltration and Activation of Neutrophils

(A) The expression of MALAT1 in each group examined by qRT-PCR. (B) The graft pulmonary vena PO_2 in each group examined by the pulmonary venous blood gas analysis. (C) The pathological changes of lung tissues in each group examined by H&E staining (scale bars, 25 μm; original magnification ×400). (D) The infiltration of macrophages (F4/80) in lung tissues in each group detected by IHC (scale bars, 50 μm; original magnification ×200). (E) The W/D ratio of lung tissues in each group. (F) The expression level of IL-8 in peripheral blood in each group determined by ELISA. (G) The expression of MPO in each group examined by IHC, with the number of neutrophils counted (scale bars, 50 μm; original magnification ×200). $^*p < 0.05$ versus the sh-NC group. All experiments were repeated three times. n = 10 in each group. Measurement data were expressed as mean ± SD. Comparisons among multiple groups were analyzed using one-way ANOVA, followed by Tukey's post hoc test. IHC, immunohistochemistry; IL-8, interleukin-8; LTIR, lung transplant ischemia-reperfusion; MALAT1, metastasis-associated lung adenocarcinoma transcript 1; MPO, myeloperoxidase; PO_2, partial pressure of oxygen; W/D, weight/dry.

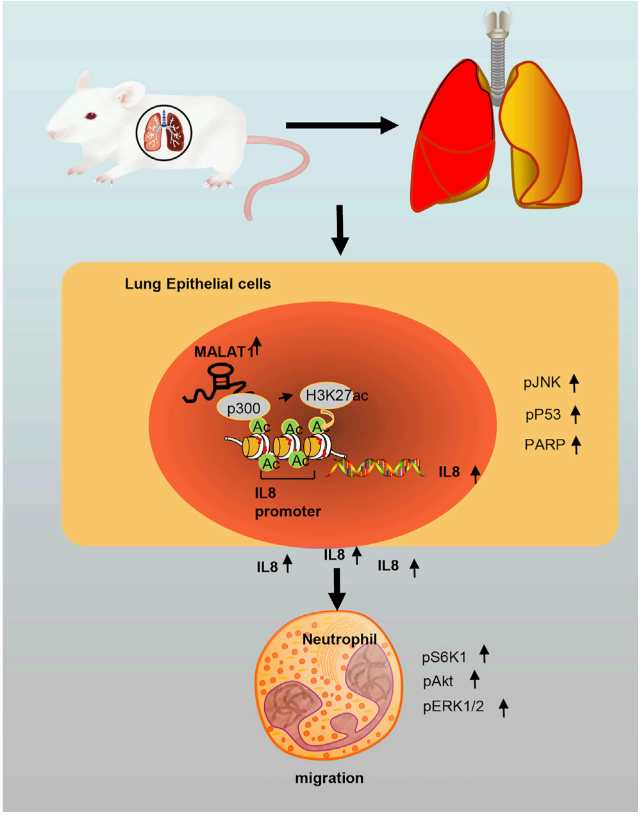

Figure 7. Proposed Molecular Mechanisms Involved in Regulation of MALAT1 in the Inflammatory Injury after LTIR

MALAT1 is highly expressed in lung tissues of LTIR rats, and enriched the H3K27ac in the IL-8 promoter region and promoted IL-8 transcription though recruiting p300, ultimately promoting the chemotaxis of neutrophils and leading to the inflammatory injury of LTIR. Our study demonstrated that silencing of MALAT1 alleviated the inflammatory injury after LTIR through downregulating IL-8 and inhibiting the infiltration of neutrophils. IL-8, interleukin-8; LTIR, lung transplant ischemia-reperfusion; MALAT1, metastasis-associated lung adenocarcinoma transcript 1.

All of these findings, together with the results in the present study, suggested that MALAT1 and IL-8 were involved in LTIR injury, and aberrant expression of MALAT1 could regulate cell apoptosis.

Furthermore, we found that MALAT1 promoted the chemotaxis of neutrophils through regulating p300-dependent IL-8. Importantly, silencing of MALAT1 could inhibit the infiltration and activation of neutrophils, thus reducing the inflammation injury after LTIR, as evidenced by the decreased expression of IL-8, p-S6K1, p-AKT, and p-ERK1/2, and reduced activity of MPO and extracellular ROS. Previously, a number of lncRNAs including MALAT1 were reported to strongly connect with the gene expression of neutrophil-enriched cell fractions.[32] lncRNA has also been demonstrated to serve as a promising therapeutic target for inflammation-related disease characterized by abnormal short-lived myeloid cells including neutrophils,[33] and MALAT1 was found to interact with NF-κB in the nucleus, thus inhibiting its DNA-binding activity and consequently reducing the generation of inflammatory factors.[24] The inhibitory effect of knockdown of MALAT1 on lung injury has been reported in several studies; for instance, silencing of MALAT1 could regulate the progression of acute lung injury through NF-κB and p38 MAPK pathways, and could also inhibit inflammation by upregulating miR-146a in lipopolysaccharide-induced acute lung injury.[34,35] Besides, upregulation of IL-8 could induce negative graft function, as well as increase mortality after lung transplantation, whereas inhibition of IL-8 by administration of mesenchymal stem cells (MSCs) improves the graft function after transplantation.[36] Therefore, these results, together with our results, supported the conclusion that knockdown of MALAT1 repressed the inflammation injury after LTIR.

In conclusion, this study demonstrated that silencing of MALAT1 could ameliorate inflammatory injury following lung transplantation by suppressing chemotaxis of neutrophils by p300-mediated enrichment of H3K27ac in an IL-8 promoter region, which provides novel insights for future therapy for LTIR injury (Figure 7).

MATERIALS AND METHODS

Ethics Statement

All animal experiments were performed with the approval of the Guide for the Care and Use of Laboratory Animals by Henan Provincial People's Hospital.

Cell Culture and Transfection

Human pulmonary epithelial cells BEAS-2B purchased from American Type Culture Collection (ATCC, MD, USA) (https://www.atcc.org/) were maintained in low-sugar DMEM (GIBCO, Carlsbad, CA, USA) containing 10% fetal bovine serum (FBS; 10100147; GIBCO BRL/Invitrogen, CA, USA) at 37°C with 5% CO_2. When cells reach 50% confluence, 10 μmol/L C646 (methyltransferase inhibitor, ab142163; Abcam, Bridge, UK) was used to inhibit methyltransferase in cells.

For transfection, LV5-GFP (lentiviral vector carrying overexpressing gene) and pSIH1-H1-copGFP (lentiviral vector carrying shRNA fluorescent expression and silenced gene) were constructed. The oligonucleotides of MALAT1 shRNA, p300 shRNA, and shRNA negative control (sh-NC) were purchased from Shanghai GenePharma (Shanghai, China). The lentiviral vectors and plasmids were transfected into 293T cells using Lip2000 according to the manufacturer's protocol. After 48 h, the supernatant was collected and centrifuged to collect the viral particles. Next, cells were inoculated into a six-well plate at a density of 5×10^4 cells/mL and infected with blank, sh-NC, overexpressing (oe)-NC, sh-MALAT1, oe-MALAT1, sh-p300, oe-p300, sh-MALAT1 + oe-NC, and sh-MALAT1 + oe-p300, respectively. After 48 h, the expression of GFP was observed under a fluorescence microscope. Besides, qRT-PCR was applied to verify the efficiency of the transfection. The experiment was repeated three times.

Isolation and Culturing of Neutrophils

Neutrophils were isolated using Histopaque 1119/1077 (Sigma-Aldrich, Poole, UK) according to the manufacturer's instruction. In

brief, peripheral venous blood was collected from healthy volunteers and then mixed with 3.2% sodium citrate solution, followed by density gradient centrifugation. Then, the neutrophils were re-suspended in PBS containing 0.5% BSA and lysed by cold MilliQ to remove infected red blood cells.

Establishment of the LTIR Rat Model

A total of 30 male Sprague-Dawley rats (aged 10–12 weeks with weight of 230–270 g) were purchased from Shanghai Laboratory Animal Centre, Chinese Academy of Sciences (Shanghai, China). All rats were housed in a specific pathogen-free (SPF) condition and fasted for 12 h before the experiments. To establish the LTIR model, the rats were randomly assigned into blank, sham, and LTIR groups (n = 10 in each group). The LTIR rat model was established according to a previous study.[1] Eight hours after lung transplant, the rats were euthanized to collect the pulmonary venous blood and lung tissues.

Function Evaluation of Lung Transplant

The function of lung transplant was evaluated by the pulmonary venous blood gas analysis using the iSTAT portable clinical analyzer (iSTAT, East Windsor, NJ, USA) according to previous studies.[1,5]

H&E Staining

The left lung tissues of rats in different groups were collected, fixed in 10% neutral formalin, and then embedded in paraffin and dewaxed by xylene. Then, the tissue sections were stained by hematoxylin, washed by distilled water, immersed in 95% ethanol, stained by eosin, hydrated by gradient ethanol, cleared by xylene, air-dried, and mounted by neutral balsam. The staining was observed under an optical microscope to determine the pathological change of lung injury.

Lung Wet/Dry (W/D) Ratio

The wet/dry ratio of the lung was determined to quantify the level of pulmonary edema. The collected lungs were weighed and then baked at 80°C for 48 h; the W/D ratio was calculated using the weights before and after drying.

ELISA

After 48 h of treatment, the cells were lysed with 60 μL western and IP cell lysate buffer (Beyotime Biotechnology, Shanghai, China), and supernatant was collected. Then, total protein concentration was determined according to the instructions of the bicinchoninic acid (BCA) kit (Beyotime Biotechnology, Shanghai, China). The absorbance (A) value at 562 nm was measured using an ELISA instrument (Vafioskan Flash; Thermo Fisher Scientific, MA, USA); then the concentrations of interleukin-6 (IL-6), IL-8, IL-4, and IL-10 were tested.

Immunohistochemistry (IHC)

IHC was conducted using the HistostainTMSP-9000 IHC staining kit (Zymed Laboratories, San Diego, CA, USA). In brief, the left lung tissues from rats were fixed by 4% paraformaldehyde, embedded by paraffin, cut into 4-μm serial sections, dewaxed, antigen repaired under microwave heating, and then blocked by normal goat serum and incubated with primary antibodies rabbit anti-F4/80 (ab111101, 1:100) and rabbit anti-myeloperoxidase (MPO) (ab9535, 1:100) at 4°C overnight. The next day, the sections were incubated with secondary antibody rat anti-rabbit (ab6728, 1:1,000) at 37°C for 30 min and, developed by 3,3′-diaminobenzidine (DAB), counterstained by hematoxylin for 1 min and mounted by balsam. Five representative fields were selected for observing and counting cells under an optical microscope (Nikon Corporation, Tokyo, Japan), and the cells with brown and yellow cytoplasm were considered positive cells.

Terminal Deoxynucleotidyl Transferase (TdT) dUTP Nick-End Labeling (TUNEL) Assay

A terminal deoxynucleotidyl transferase (TdT) dUTP nick-end labeling (TUNEL) apoptosis detection kit (Millipore, Billerica, MA, USA) was used to detect the cell apoptosis in lung tissues according to a previous study.[37] Ten fields in each section were randomly selected, and the positive cells and podocytes were counted. Cells with brown-yellow nucleus were apoptotic positive cells and with a blue nucleus were normal cells. The apoptotic rate of cells was expressed as: number of brown-yellow cells/number of blue cells $\times$ 100%.

Fluorescence *In Situ* Hybridization (FISH)

Fluorescence *in situ* hybridization (FISH) was used to detect the expression of MALAT1 in lung tissues. The transplanted lung tissues were fixed in paraformaldehyde, embedded in paraffin, and sectioned into slices. The sections were detached by pepsin to reduce the contamination of RNase. The slides were blocked by Dako and then incubated with digoxin-labeled MALAT1 probe (EXIQON, Vedback, Denmark) for detecting MALAT1. The sections were incubated with secondary antibody (ab6728, 1:1,000; Abcam, Cambridge, UK) at room temperature, stained by Prolong Gold Anti-fade Reagent diamidino-phenyl-indole (DAPI; Invitrogen, Carlsbad, CA, USA), and then photographed using Leica DFC360 FX Camera under a Leica DMRA fluorescence microscope (Leica, Wetzlar, Germany). Lastly, the images were assembled using Adobe Illustrator.

Establishment of the LTIR Cell Model

BEAS-2B cells were cultured at 37°C with 5% CO_2 until cell fusion and then cultured with icy lung preservation liquid Perfadex (Vitrolife, Englewood, CO, USA) to establish the model of LTIR cells according to the previous study.[38]

RNA Isolation and Quantitation

Total RNA was extracted from cell and tissue samples using TRIzol (Invitrogen, Carlsbad, CA, USA; Thermo Fisher Scientific, MA, USA), the quantity and concentration of RNA were determined using UV spectrophotometer (ND-1000; NanoDrop Technologies, Wilmington, DE, USA). Then 400 ng total RNA was reversely transcribed into cDNA using the PrimeScript RT Reagent Kit (Takara Biotechnology, Dalian, China). qRT-PCR was conducted according to the instructions of SYBR Premix Ex Taq II (Tli RNaseH Plus) kit (Takara Bio, Otsu, Shiga, Japan) on the Thermal Cycler Dice Real Time System Instrument (TP800; Takara Bio, Otsu, Shiga, Japan).

Table 1. The Primer Sequences for qRT-PCR

Gene	Sequences (5′-3′)
MALAT1 (Homo)	F: 5′-TCTTAGAGGGTGGGCTTTTGTT-3′
	R: 5′-CTGCATCTAGGCCATCATACTG-3′
MALAT1 (Mus)	F: 5′-TAAGCGCTTGCCTCTGTCTT-3′
	R: 5′-CACCTGCATTCTGTGTGGTC-3′
p300	F: 5′-GACCCTCAGCTTTTAGGAATCC-3′
	R: 5′-TGCCGTAGCAACACAGTGTCT-3′
IL-8 (Homo)	F: 5′-CTCTGTGGTATCCAAGAATCAGTGA-3′
	R: 5′-TATTGCATCTGGCAACCCTACA-3′
IL-8 (Mus)	F: 5′-CCTAGGCATCTTCGTCCGTC-3′
	R: 5′-CAGAAGCTTCATTGCCGGTG-3′
GAPDH (Homo)	F: 5′-GCAAATTCCATGGCACCGT-3′
	R: 5′-TCGCCCCACTTGATTTTGGAGG-3′
GAPDH (Mus)	F: 5′-AAGGTCATCCCAGAGCTGAA-3′
	R: 5′-GCCATGAGGTCCACCACCCT-3′

F, forward; GAPDH, glyceraldehyde-3-phosphate dehydrogenase; IL-8, interleukin-8; MALAT1, metastasis associated lung adenocarcinoma transcript 1; R, reverse.

The primers were synthesized by Guangzhou RiboBio (Guangzhou, China) (Table 1). With glyceraldehyde-3-phosphate dehydrogenase (GAPDH) used as the internal reference, the expression of genes was calculated by means of the relative quantification $2-\Delta\Delta Ct$ method.

Western Blot Analysis

The pulmonary epithelial tissues or neutrophils were lysed by western cell lysate buffer (C0481; Sigma-Aldrich Chemical Company, St. Louis, MO, USA). The protein concentration was determined using BCA kit (Beyotime Biotechnology, Shanghai, China). Equal amounts of proteins (20 mg) were separated by 10% SDS-PAGE and transferred onto the polyvinylidene fluoride (PVDF) membrane (Millipore, Billerica, MA, USA). After blocking with 5% dried skimmed milk for 1 h, the membrane was incubated with the following primary antibodies: rabbit antibodies JNK (2305, 1:1,000), p-JNK (4668, 1:1,000), P53 (2527, 1:1,000), p-P53 (9284, 1:1,000), poly-ADP-ribose polymerase (PARP) (9532, 1:1,000), S6K1 (2708, 1:1,000), p-S6K1 (97596, 1:1,000), Akt (4685, 1:1,000), p-Akt (4060, 1:2,000), Erk1/2 (4695, 1:1,000), and p-Erk1/2 (4370, 1:2,000) at 4°C overnight. All of the antibodies were purchased from Cell Signaling Technology (Beverly, MA, USA). After washing three times, the membrane was incubated with horseradish peroxidase-labeled secondary antibody (ab6728, 1:1,000; Abcam, Cambridge, UK) at room temperature for 1 h and then developed by electrochemiluminescence (ECL) (Baoman Biotechnology, Shanghai, China). GAPDH was used as an internal reference.

Flow Cytometry

The flow cytometry (Thermo Fisher Scientific, MA, USA) was used to detect the apoptosis of pulmonary epithelial cells. The pulmonary epithelial cells at passages 3–6 were used and transfected with sh-MALAT1 and oe-MALAT1. Cell suspension was successively stained by 5 μL Annexin V-fluorescein isothiocyanate (FITC) and propidium iodide (PI) dye. The apoptosis was analyzed by flow cytometry; the ratio of cell apoptosis was obtained from the ratio of cells with green fluorescence to the cells with red fluorescence.

Table 2. The Primer Sequences for the IL-8 Promoter

Gene	Primer Sequences (5′-3′)
IL-8	F: 5′-AAGTGTGATGACTCAGGTTTGC-3′
	R: 5′-GAAGCTTGTGTGCTCTGCGT-3′

F, forward; IL-8, interleukin-8; R, reverse.

Double FISH

Double FISH assay was conducted to detect the intranuclear localization of MALAT1 and p300 as previously described.[39] In brief, the pulmonary epithelial cells were seeded in a 24-well plate at the concentration of 5×10^3 cells/well for 24 h and fixed in 4% paraformaldehyde at room temperature for 10 min. The cells were then washed with PBS containing 0.5% Triton X-100, blocked with pre-hybridization solution at room temperature, and incubated with MALAT1 probe and primary antibody to p300 (Sc-32244, 1:500, Santa Cruz, USA) at room temperature overnight. After incubation with secondary antibody (8890, 1:500, Cell Signaling), the cells were counterstained with DAPI and observed using a Nikon Eclipse E800 confocal microscope.

Chromatin Immunoprecipitation (ChIP)

ChIP was conducted to examine the enrichment of p300 and H3K27ac on the IL-8 promoter region using the EZ-Magna ChIP TMA kit (Millipore, Billerica, MA, USA). The pulmonary epithelial cells were cross-linked with 1% formaldehyde for 10 min and then lysed in lysis buffer containing protease inhibitors. Then, the supernatant was collected and incubated with antibodies. The precipitates were washed, eluted by ChIP Wash Buffer, and de-crosslinked. After recovery of DNA, qRT-PCR was applied to quantify the IL-8 promoter. The primer sequences of the IL-8 promoter are shown in Table 2.

RNA Immunoprecipitation (RIP)

RIP was applied to verify the interaction between p300 and MALAT1 using the Magna RIP TM RNA-Binding Protein Immunoprecipitation Kit (Millipore, Billerica, MA, USA). In brief, the pulmonary epithelial cells were lysed. A total of 100 μL cell extract was incubated with 900 μL RIP buffer containing magnetic bead coated by p300 rabbit antibody (ab14984, 1:50; Abcam, Cambridge, UK) or immunoglobulin G (IgG) for 3 h at 4°C. Beads were washed three times with lysis buffer, and RNA was extracted by addition of TRIzol to the beads. Finally, qPCR was performed to detect the MALAT1.

RNA Pull-Down

RNA pull-down was employed to examine the association between p300 and MALAT1. The purified RNA 3′ end of MALAT1was labeled

by biotin RNA-labeled mixture (Ambion; Thermo Fisher Scientific, Waltham, MA, USA). A total of 400 ng biotinylated RNA in 500 μL RIP buffer was incubated with cell lysate. Western blot was performed to detect the expression of p300.

Immunoprecipitation (IP)

To determine the interaction between p300 and IL-8, we conducted IP to detect whether IL-8 antibody could immunoprecipitate p300; carbohydrate-binding protein (CBP) that interacted with p300 was used as a positive control, and immunoglobulin G (IgG) was used as a negative control. Cells transfected with pCDNA3-HA-p300 were lysed, and protein samples were collected. Anti-IL-8 antibody and Sepharose beads were incubated with protein samples overnight. The IP beads were washed three times with cold lysis buffer. The binding proteins were eluted and separated by SDS-PAGE and then verified by western blot analysis.

Transwell Assay

The neutrophils were isolated from the whole blood of healthy volunteers; then the starved cells were pre-activated by formyl-leucyl-methionine peptide (fLMP) (Nova Biochem, Darmstadt, Germany) for 10 min and treated by inhibitor or IL-8 neutralizing antibody for 10 min. A total of 1.5×10^5 cells were re-suspended and placed in the Transwell migration chamber (3 mm; BD Biosciences, Franklin Lakes, NJ, USA) supplemented with serum-free medium containing 0.1% BSA. The serum-free DMEM was added into the lower chamber of the Transwell. After removal of the un-migrated cells, the migrated cells were stained by Hema-3 (Fisher Scientific, Pittsburgh, PA, USA) and counted.

Establishment of the LTIR Rat Model with Depletion of MALAT1

A total of 50 clean Sprague-Dawley male rats weighing 230–270 g (provided by Shanghai Laboratory Animal Center, Chinese Academy of Sciences, Shanghai, China) were used in this study. The cuff technique of artery, vein, and bronchus was used to establish the orthotopic left lung transplant model in rats. After lung transplant, 1×10^7 adenoviruses containing sh-NC or sh-MALAT1were injected into rats via tail vein, and peripheral venous blood was collected at corresponding time points; then the rats were euthanized by intravenous injection of 3% pentobarbital sodium (P3761; Sigma-Aldrich Chemical Company, St. Louis, MO, USA) (three times concentration). After 2 weeks, the lung tissues of rats were obtained to examine the expression of related factors.

Detection of MPO by Chromatometry

Chromatometry was employed to detect the activity of MPO according to the instructions of the MPO detection kit (K747-100; BioVision, San Francisco, CA, USA) based on the previous study.[40]

Statistical Analysis

All data were processed by SPSS 21.0 statistical software (IBM, Armonk, NY, USA). Measurement data were expressed as mean ± SD. Comparisons among multiple groups were analyzed using one-way ANOVA followed by Tukey's post hoc test. The data obtained by multiple measurements at different time points were analyzed using repeated-measures ANOVA followed by Bonferroni post hoc test. $p < 0.05$ was considered statistically significant.

SUPPLEMENTAL INFORMATION

Supplemental Information can be found online at https://doi.org/10.1016/j.omtn.2019.05.009.

AUTHOR CONTRIBUTIONS

J.L. and Z.H. designed the study. Z.C. and J.L. collated the data, designed and developed the database, carried out data analyses, and produced the initial draft of the manuscript. Q.Z. and L.W. contributed to drafting the manuscript. All authors have read and approved the final submitted manuscript.

CONFLICTS OF INTEREST

The authors declare no competing interests.

ACKNOWLEDGMENTS

We acknowledge and appreciate our colleagues for their valuable efforts and comments on this paper. This study was supported by the "23456 Talent Project" of Henan Provincial People's Hospital.

REFERENCES

1. Mallavia, B., Liu, F., Sheppard, D., and Looney, M.R. (2016). Inhibiting Integrin αvβ5 Reduces Ischemia-Reperfusion Injury in an Orthotopic Lung Transplant Model in Mice. Am. J. Transplant. *16*, 1306–1311.

2. Watanabe, T., Hoshikawa, Y., Ishibashi, N., Suzuki, H., Notsuda, H., Watanabe, Y., Noda, M., Kanehira, M., Ohkouchi, S., Kondo, T., and Okada, Y. (2017). Mesenchymal stem cells attenuate ischemia-reperfusion injury after prolonged cold ischemia in a mouse model of lung transplantation: a preliminary study. Surg. Today *47*, 425–431.

3. Merry, H.E., Wolf, P.S., Fitzsullivan, E., Keech, J.C., and Mulligan, M.S. (2010). Lipopolysaccharide pre-conditioning is protective in lung ischemia-reperfusion injury. J. Heart Lung Transplant. *29*, 471–478.

4. Ailawadi, G., Lau, C.L., Smith, P.W., Swenson, B.R., Hennessy, S.A., Kuhn, C.J., Fedoruk, L.M., Kozower, B.D., Kron, I.L., and Jones, D.R. (2009). Does reperfusion injury still cause significant mortality after lung transplantation? J. Thorac. Cardiovasc. Surg. *137*, 688–694.

5. Kawamura, T., Huang, C.S., Tochigi, N., Lee, S., Shigemura, N., Billiar, T.R., Okumura, M., Nakao, A., and Toyoda, Y. (2010). Inhaled hydrogen gas therapy for prevention of lung transplant-induced ischemia/reperfusion injury in rats. Transplantation *90*, 1344–1351.

6. Chen, F., and Date, H. (2015). Update on ischemia-reperfusion injury in lung transplantation. Curr. Opin. Organ Transplant. *20*, 515–520.

7. Gutschner, T., Hämmerle, M., Eissmann, M., Hsu, J., Kim, Y., Hung, G., Revenko, A., Arun, G., Stentrup, M., Gross, M., et al. (2013). The noncoding RNA MALAT1 is a critical regulator of the metastasis phenotype of lung cancer cells. Cancer Res. *73*, 1180–1189.

8. Lai, M.C., Yang, Z., Zhou, L., Zhu, Q.Q., Xie, H.Y., Zhang, F., Wu, L.M., Chen, L.M., and Zheng, S.S. (2012). Long non-coding RNA MALAT-1 overexpression predicts tumor recurrence of hepatocellular carcinoma after liver transplantation. Med. Oncol. *29*, 1810–1816.

9. Kölling, M., Genschel, C., Kaucsar, T., Hübner, A., Rong, S., Schmitt, R., Sörensen-Zender, I., Haddad, G., Kistler, A., Seeger, H., et al. (2018). Hypoxia-induced long non-coding RNA Malat1 is dispensable for renal ischemia/reperfusion-injury. Sci. Rep. *8*, 3438.

10. Yu, S.Y., Dong, B., Zhou, S.H., and Tang, L. (2017). LncRNA MALAT1: A potential regulator of autophagy in myocardial ischemia-reperfusion injury. Int. J. Cardiol. *247*, 25.

11. Zhao, Z.H., Hao, W., Meng, Q.T., Du, X.B., Lei, S.Q., and Xia, Z.Y. (2017). Long non-coding RNA MALAT1 functions as a mediator in cardioprotective effects of fentanyl in myocardial ischemia-reperfusion injury. Cell Biol. Int. *41*, 62–70.

12. Chen, H., Wang, X., Yan, X., Cheng, X., He, X., and Zheng, W. (2018). LncRNA MALAT1 regulates sepsis-induced cardiac inflammation and dysfunction via interaction with miR-125b and p38 MAPK/NFκB. Int. Immunopharmacol. *55*, 69–76.

13. Pigossi, S.C., Anovazzi, G., Finoti, L.S., de Medeiros, M.C., Mayer, M.P.A., Rossa Junior, C., and Scarel-Caminaga, R.M. (2019). Functionality of the Interleukin 8 haplotypes in lymphocytes and macrophages in response to gram-negative periodontopathogens. Gene *689*, 152–160.

14. Pine, S.R., Mechanic, L.E., Enewold, L., Chaturvedi, A.K., Katki, H.A., Zheng, Y.L., Bowman, E.D., Engels, E.A., Caporaso, N.E., and Harris, C.C. (2011). Increased levels of circulating interleukin 6, interleukin 8, C-reactive protein, and risk of lung cancer. J. Natl. Cancer Inst. *103*, 1112–1122.

15. Moreno, I., Mir, A., Vicente, R., Pajares, A., Ramos, F., Vicente, J.L., and Barbera, M. (2008). Analysis of interleukin-6 and interleukin-8 in lung transplantation: correlation with nitric oxide administration. Transplant. Proc. *40*, 3082–3084.

16. Neri, F., Puviani, L., Tsivian, M., Prezzi, D., Pacilé, V., Cavallari, G., Bertelli, R., Bianchi, E., Piras, G.L., Pariali, M., et al. (2007). Protective effect of an inhibitor of interleukin-8 (meraxin) from ischemia and reperfusion injury in a rat model of kidney transplantation. Transplant. Proc. *39*, 1771–1772.

17. Banerjee, S., Arif, M., Rakshit, T., Roy, N.S., Kundu, T.K., Roy, S., and Mukhopadhyay, R. (2012). Structural features of human histone acetyltransferase p300 and its complex with p53. FEBS Lett. *586*, 3793–3798.

18. Bartling, T.R., and Drumm, M.L. (2009). Loss of CFTR results in reduction of histone deacetylase 2 in airway epithelial cells. Am. J. Physiol. Lung Cell. Mol. Physiol. *297*, L35–L43.

19. Wan, G., Hu, X., Liu, Y., Han, C., Sood, A.K., Calin, G.A., Zhang, X., and Lu, X. (2013). A novel non-coding RNA lncRNA-JADE connects DNA damage signalling to histone H4 acetylation. EMBO J. *32*, 2833–2847.

20. Kaido, T., and Uemoto, S. (2010). Effects of neutrophil elastase inhibitor on progression of acute lung injury after liver transplantation. Transplantation *90*, 335–337.

21. Su, X., Looney, M.R., Su, H.E., Lee, J.W., Song, Y., and Matthay, M.A. (2011). Role of CFTR expressed by neutrophils in modulating acute lung inflammation and injury in mice. Inflamm. Res. *60*, 619–632.

22. Xue, Z., Tang, Y., Dai, M., Chen, S., and Shen, N. (2017). 121 Interferon stimulated long noncoding rna lncrna-cmpk2 facilitates neutrophils interferon production by tlr7/8 agonist in sle. Lupus Sci. Med. *4* (*Suppl 1*), A55.

23. Wang, Y., Nie, W., Yao, K., Wang, Z., and He, H. (2016). Interleukin 6 induces expression of NADPH oxidase 2 in human aortic endothelial cells via long noncoding RNA MALAT1. Pharmazie *71*, 592–597.

24. Zhao, G., Su, Z., Song, D., Mao, Y., and Mao, X. (2016). The long noncoding RNA MALAT1 regulates the lipopolysaccharide-induced inflammatory response through its interaction with NF-κB. FEBS Lett. *590*, 2884–2895.

25. Laubach, V.E., and Sharma, A.K. (2016). Mechanisms of lung ischemia-reperfusion injury. Curr. Opin. Organ Transplant. *21*, 246–252.

26. Zhai, W., Li, X., Wu, S., Zhang, Y., Pang, H., and Chen, W. (2015). Microarray expression profile of lncRNAs and the upregulated ASLNC04080 lncRNA in human endometrial carcinoma. Int. J. Oncol. *46*, 2125–2137.

27. Imamura, K., Imamachi, N., Akizuki, G., Kumakura, M., Kawaguchi, A., Nagata, K., Kato, A., Kawaguchi, Y., Sato, H., Yoneda, M., et al. (2014). Long noncoding RNA NEAT1-dependent SFPQ relocation from promoter region to paraspeckle mediates IL8 expression upon immune stimuli. Mol. Cell *53*, 393–406.

28. Zou, A., Liu, R., and Wu, X. (2016). Long non-coding RNA MALAT1 is up-regulated in ovarian cancer tissue and promotes SK-OV-3 cell proliferation and invasion. Neoplasma *63*, 865–872.

29. Chen, R., Liu, Y., Zhuang, H., Yang, B., Hei, K., Xiao, M., Hou, C., Gao, H., Zhang, X., Jia, C., et al. (2017). Quantitative proteomics reveals that long non-coding RNA MALAT1 interacts with DBC1 to regulate p53 acetylation. Nucleic Acids Res. *45*, 9947–9959.

30. Tano, K., Onoguchi-Mizutani, R., Yeasmin, F., Uchiumi, F., Suzuki, Y., Yada, T., and Akimitsu, N. (2018). Identification of Minimal *p53* Promoter Region Regulated by MALAT1 in Human Lung Adenocarcinoma Cells. Front. Genet. *8*, 208.

31. Cai, C., Qiu, J., Qiu, G., Chen, Y., Song, Z., Li, J., and Gong, X. (2017). Long non-coding RNA MALAT1 protects preterm infants with bronchopulmonary dysplasia by inhibiting cell apoptosis. BMC Pulm. Med. *17*, 199.

32. Pellegrina, D.V.D.S., Severino, P., Barbeiro, H.V., de Souza, H.P., Machado, M.C.C., Pinheiro-da-Silva, F., and Reis, E.M. (2017). Insights into the Function of Long Noncoding RNAs in Sepsis Revealed by Gene Co-Expression Network Analysis. Noncoding RNA *3*, e5.

33. Kotzin, J.J., Spencer, S.P., McCright, S.J., Kumar, D.B.U., Collet, M.A., Mowel, W.K., Elliott, E.N., Uyar, A., Makiya, M.A., Dunagin, M.C., et al. (2016). The long non-coding RNA Morrbid regulates Bim and short-lived myeloid cell lifespan. Nature *537*, 239–243.

34. Dai, L., Zhang, G., Cheng, Z., Wang, X., Jia, L., Jing, X., Wang, H., Zhang, R., Liu, M., Jiang, T., et al. (2018). Knockdown of LncRNA MALAT1 contributes to the suppression of inflammatory responses by up-regulating miR-146a in LPS-induced acute lung injury. Connect. Tissue Res. *59*, 581–592.

35. Li, H., Shi, H., Ma, N., Zi, P., Liu, Q., and Sun, R. (2018). BML-111 alleviates acute lung injury through regulating the expression of lncRNA MALAT1. Arch. Biochem. Biophys. *649*, 15–21.

36. Mordant, P., Nakajima, D., Kalaf, R., Iskender, I., Maahs, L., Behrens, P., Coutinho, R., Iyer, R.K., Davies, J.E., Cypel, M., et al. (2016). Mesenchymal stem cell treatment is associated with decreased perfusate concentration of interleukin-8 during ex vivo perfusion of donor lungs after 18-hour preservation. J. Heart Lung Transplant. *35*, 1245–1254.

37. Chuang, L.H., Wu, A.L., Wang, N.K., Chen, K.J., Liu, L., Hwang, Y.S., Yeung, L., Wu, W.C., and Lai, C.C. (2018). The intraocular staining potential of anthocyanins and their retinal biocompatibility: a preclinical study. Cutan. Ocul. Toxicol. *37*, 359–366.

38. Kim, H., Zhao, J., Zhang, Q., Wang, Y., Lee, D., Bai, X., Turrell, L., Chen, M., Gao, W., Keshavjee, S., and Liu, M. (2016). δV1-1 Reduces Pulmonary Ischemia Reperfusion-Induced Lung Injury by Inhibiting Necrosis and Mitochondrial Localization of PKCδ and p53. Am. J. Transplant. *16*, 83–98.

39. Chen, Z.Z., Huang, L., Wu, Y.H., Zhai, W.J., Zhu, P.P., and Gao, Y.F. (2016). LncSox4 promotes the self-renewal of liver tumour-initiating cells through Stat3-mediated Sox4 expression. Nat. Commun. *7*, 12598.

40. Namkoong, H., Ishii, M., Fujii, H., Yagi, K., Asami, T., Asakura, T., Suzuki, S., Hegab, A.E., Kamata, H., Tasaka, S., et al. (2018). Clarithromycin expands CD11b+Gr-1+ cells via the STAT3/Bv8 axis to ameliorate lethal endotoxic shock and post-influenza bacterial pneumonia. PLoS Pathog. *14*, e1006955.

OMTN, Volume *18*

Supplemental Information

Silencing of lncRNA MALAT1 Prevents Inflammatory Injury after Lung Transplant Ischemia-Reperfusion by Downregulation of IL-8 via p300

Li Wei, Jiwei Li, Zhijun Han, Zhong Chen, and Quan Zhang

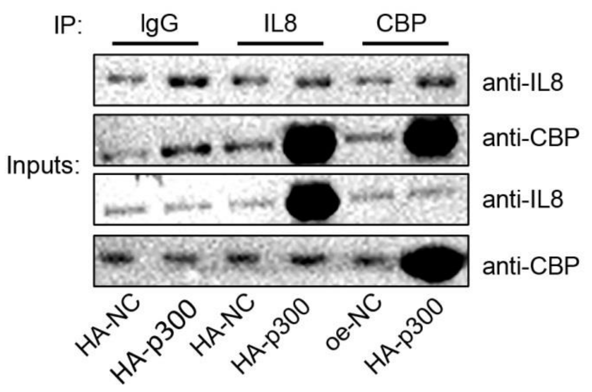

Supplementary Figure 1 The interaction between p300 and IL8 by IP; CBP was used as the positive control while IgG as the negative control.
